# Practical Points

**Published:** 1907-11-30

**Authors:** 


### PRACTICAL POINTS.

### HOSPITALS AND MEDICAL TREATMENT. FEES PROVIDED UNDER THE WORKMEN'S COMPENSATION ACT.

A SUBSCRIBER to THE HOSPITAL writes: A question has arisen in connection with our local hospital, of which I am a director, and I shall be very glad if you can assist me thereon.

The hospital is supported by voluntary contributions, and patients are admitted by notes of recommendation, obtainable from subscribers.

It appears that in certain instances injured workmen furnished with notes have been received at the hospital and treated there, and afterwards, when they have made claims against their employers under the Workmen's Compensation Act, the honorary surgeons on the hospital staff who have attended them have, when requested by the employers, without the consent of their late patients, the workmen, given statements to the employers of what they know of the cases.

A representation has recently been made by a deputation from one of the bodies of workmen contributing to the income of the hospital that the men consider that this giving of statements to employers is not right or fair towards them.

I should say, as helping to explain the feeling of the workmen, that of the total income of the hospital (about £6,000) about £1,600 is received from contributions by working men collected through a Committee appointed by themselves for the purpose, their Committee receives notes of recommendation in respect of such contributions, and these notes are largely used by the workmen. Many of the workmen in consequence of such contributions consider (I think erroneously) that when injured they have a claim amounting to a right to receive treatment at the hospital.

It seems to me that an important question of principle is raised—namely: Does the fact of the patient paying nothing, and the surgeon, as an honorary surgeon to the hospital giving his services, free the surgeon from the obligation, which I understand that he is under, towards his own paying patients, not to disclose (except, of course, in the witness-box) any information obtained from the relationship of surgeon and patient.

The legal authorities seem to show that a surgeon's obligations towards a hospital patient do not differ from those towards a private paying patient so far as the employment of reasonable and proper professional skill and care is [illegible] cerned, and I fail at present to see why this obligation s[illegible] not extend to the non-disclosure of information.

Apart from the principle involved, the hospital, of co[illegible] does not on the one hand wish to offend a considerable [illegible] of its contributors nor on the other hand to depriv[illegible] honorary staff of fees for statements to which they are l[illegible] mately entitled.

I shall be very glad if you can assist me on the poin[illegible] write, of course, non-officially and for my private info[illegible] tion; and, as the matter is under the consideration [illegible] Committee of the directors, may I ask you at present to [illegible] with it anonymously.

[The questions raised depend largely upon the constitu[illegible] and methods of the particular hospital. If the by-laws [illegible] regulations provide for the admission of paying pati[illegible] there need be no objection to the Committee entering [illegible] an arrangement to charge so much per week for the tr[illegible] ment of patients in the pay wards, and to set aside a [illegible] centage of such payments for the remuneration of the mec[illegible] staff. No doubt the effect of the Workmen's Compensa[illegible] Act will be to cause a rearrangement of the existing relat[illegible] between hospitals and patients of the artisan class who s[illegible] admission to them and the large body of the medical [illegible] fession. It is perfectly clear that if the employers ar[illegible] provide the cost of medical service, that cost, though it [illegible] be paid in the first instance to the patient, will put [illegible] patient in the position which he does not now prope[illegible] occupy—of being able to pay for his medical attenda[illegible] In such circumstances it should be the care of the hosp[illegible] to see that the money should be properly applied to de[illegible] the cost of medical attendance as thus supplied, so a[illegible] secure that it does not accrue to the patient who is abl[illegible] get all the medical care that he needs from the hospit[illegible] present without payment of any kind. No right-thin[illegible] member of the artisan class would permit himself to re[illegible] money for the cost of his medical care and treatment [illegible] not hand that money on to remunerate those who [illegible] supplied him with these essential aids to recovery.—THE HOSPITAL.]

